# Current Overview of CDKL-5 Deficiency Disorder Treatment

**DOI:** 10.3390/pediatric16010002

**Published:** 2024-01-03

**Authors:** Giovanni Battista Dell’Isola, Katherin Elizabeth Portwood, Kirsten Consing, Antonella Fattorusso, Arnaldo Bartocci, Pietro Ferrara, Giuseppe Di Cara, Alberto Verrotti, Mauro Lodolo

**Affiliations:** 1 Department of Pediatrics, University of Perugia, 06129 Perugia, Italy; antonella.fattorusso@ospedale.perugia.it (A.F.); giuseppe.dicara@unipg.it (G.D.C.); alberto.verrottidipianella@unipg.it (A.V.); 2 Shands Children’s Hospital, Department of Child Neurology, University of Florida, Gainesville, FL 32608, USA; kportwood@ufl.edu (K.E.P.); consing.kirsten@ufl.edu (K.C.); lodolom@ufl.edu (M.L.); 3 Neurophysipathology Service, Villa Margherita, 01027 Montefiascone, Italy; abartox@gmail.com; 4 Unit of Pediatrics, Campus Bio-Medico University, 00128 Rome, Italy; p.ferrara@unicampus.it

CDKL5 deficiency disorder (CDD) is a complex of clinical symptoms resulting from the presence of non-functional or absent CDKL5 protein, a serine–threonine kinase involved in neural maturation and synaptogenesis [1]. Although originally considered a variant of Rett syndrome because of clinical similarities with patients harboring pathogenic MECP2 variants, CDD is now considered an independent clinical entity caused by a pathogenic mutation in the CDKL5 gene [2,3]. Early epileptic encephalopathy and severe developmental delay are the main features of CDD [4,5]. Other comorbidities are equally prominent, such as hypotonia, sleep disturbances due to obstructive airway symptoms and central apnea, movement disorders (i.e., dyskinesias, chorea, and hand stereotypies), cortical visual impairment with poor eye fixation, dysphagia, and other gastrointestinal problems [6,7,8].

The prevalence of mutations in CDKL5 is estimated at approximately 1 in 40,000–60,000 live births [9]. CDKL5 is located on the X chromosome; therefore, CDD behaves like an X-linked disorder that affects females four times more than males, as males with germline variants have no normal CDKL5 protein and may not survive fetal life [10]. 

As a developmental epileptic encephalopathy, the most prominent and presenting feature of CDD is the early onset of seizures. The median age of epilepsy onset is 6 weeks, with 90% onset by 12 months of age, according to Fehr and colleagues [2,4]. Although most seizure types can be seen in patients with CDD, epileptic spasms (with or without hypsarrhythmia) and tonic seizures with asymmetrical or focal features are the most common at the onset and over the disease course, followed by focal, myoclonic, and generalized tonic-clonic seizures [11,12]. Mixed motor and autonomic features can occur during the same seizure, making classification challenging [11].

After a variable honeymoon period in which patients can experience seizure freedom, most of them became pharmacoresistant to antiepileptic drugs. Less than 10% of patients have several episodes a month, about 12% have several episodes weekly, and up to 80% have seizures every day, up to 10–20 attacks per day [1].

Limited successful treatment methods have been described in the CDD. In a recent work by Olson et al., the authors have reported in a very explanatory table all the therapies available up to now with unfortunately rather disappointing results [13]. Only in this last year, two drugs have been added, Ganaxolone and Fenfluramine, which have opened up a new therapeutic possibility even if it still appears too early to conclude the performance of the patients treated.

Ganaxolone hydroxy-3β-methyl-5δ-pregnane-20-one) belongs to a new group of neuroactive steroids called epalons that act as positive allosteric modulators of synaptic and extrasynaptic GABA A receptors to enhance GABAergic inhibitory tone [14]. Neuroactive steroids are a class of molecules that derive from the breakdown of progesterone or deoxycorticosterone and are enriched in the nervous system. Ganaxolone is a synthetic beta-methylated analog of allopregnanolone and is a neurosteroid that exhibits potent antiepileptic effects in several animal models of epilepsy.

The recent Marigold study [15] led to the approval by the US Food and Drug Administration (FDA) of ganaxolone for treating seizures in CDD patients aged 2 years and older. In this study, 101 patients affected by CDD aged 2 to 21 years were randomly treated with ganaxolone or placebo. Patients weighing ≤28 kg were administered a maximum dose of 21 mg/kg three times daily and 600 mg three times daily for patients >28 kg. Efficacy was measured as a percentage change in median 28-day motor seizure frequency. Ganaxolone was associated with a significant reduction of CDD-associated seizure (−30.7%) compared to a 6.9% reduction in the placebo group (*p* = 0.0036). The therapy was overall well tolerated, without any difference of serious side effects compared to placebo. Somnolence was the most frequent adverse event (36% of patients).

Fenfluramine (FFA) enhances serotonin release, positively modulates sigma-1 receptors, and has potent and long-lasting efficacy in the treatment of convulsive seizures in Dravet syndrome and epileptic seizures in Lennox–Gastaut syndrome, with approval for Dravet syndrome by the FDA and the European Medicines Agency (EMA) [16]. Long-term open-label extension studies and experience in Belgium have demonstrated a sustained reduction in seizure frequency for up to 30 years in patients with Dravet syndrome, with no observations of pulmonary arterial hypertension or valvular heart disease in any patient at any time [17].

In the literature, there is only one study published in *Epilepsia* by Devinsky et al. specific to the use of FFA on patients with CDKL-5-related epilepsy [18]. In fact, such a study limited to only six patients would have demonstrated a good response on generalized tonic–clonic and tonic seizures while having little effect on spasms and myoclonic seizures. FFA was administered at 0.4 mg/kg/day or 0.7 mg/kg/day for a mean treatment duration of 5.3 months (range: 2–9 months).

Based on these data, further studies are expected to approve the use of fenfluramine in the forms of CDKL-5 epilepsy.

CDD-related epilepsy is refractory to available medications and dietary and neurostimulation therapies; however, some benefits have been studied in the highly purified cannabidiol, Epidiolex. 

An open-label study completed by Devinsky et al. is the primary resource for the benefits of Epidolex as a treatment for CDKL5 deficiency. The authors studied the effects of Epidiolex in 55 patients between the ages of 1 and 30. Initial doses of 5 mg/kg/day divided twice daily were titrated between 2 and 10 mg/kg/day to a max dose of 50 mg/kg/day divided twice daily. Devinsky’s research indicates that patients with CDD required lower doses of Epidiolex for convulsive type seizure control compared to other Epileptic Encephalopathies (EE) (Aicardi, Doose, Dup15q) [19]. The median seizure frequency of convulsive seizures decreased by 50% by week 12 with Epidiolex use, and seizure control was maintained on Epidiolex by week 48 [19]. In addition, among the four types of EE monitored, CDKL5 deficiency has the second-best responder rate to Epidiolex, with 41% at week 12 and 53% at week 48 [19]. Epidiolex also affected other medications used to control EEs. Indeed, patients already on two or more antiseizure medications reported decreased dosages of clobazam, levetiracetam, valproic acid, and rufinamide. Lamotrigine dosing remained stable, and fenfluramine dosing increased. Therefore, Epidiolex can potentially decrease the dosing of other medications used for seizure control.

The ketogenic diet (KD) is known to be a non-pharmacologic approach to treating epilepsy, consisting of a strict low-carbohydrate and high-fat diet. It is hypothesized that increased ketone body concentrations could lead to enhanced inhibitory neurotransmission and, therefore, reduced seizure frequency [20]. Constipation, vomiting, renal calculi, prolonged QTc interval, and dyslipidemia are important side effects to take into consideration before starting the KD [21]. 

As a result of the refractory epileptic nature of CDD, KD has been trialed in this population. Observational studies have shown mixed results for the KD-perceived reduction in seizure frequency and improved benefits on behavior, cognition, and quality of life [22]. In one study, 104 out of 204 individuals from the International CDKL5 Disorder Database implemented the KD, where at least half of the cohort had an initial improvement in seizure activity regarding decreased seizure frequency. Side effects from KD were reported in 31.7% of individuals. It had poor long-term efficacy, as the mean duration of the diet was about 1.4 years [23,24]. Along with other studies, there are concerns that the KD does not work well for long-term management of CDKL5-associated epilepsy [22,25]. The latest systemic review and meta-analysis study has shown that the definite responder rate of KD in CDKL5-related epilepsy was only 18%. A definite responder rate was defined as the proportion of patients with seizure frequency reduced greater than or equal to 50% from baseline after KD therapy [26].

Palliative surgeries such as vagus nerve stimulator (VNS) and corpus callosotomy have been used in CDD patients with more severe epilepsy. 

Most studies on the efficacy of VNS have been in retrospective case series and caregiver surveys. One study has shown improvement in seizure activity for over two-thirds of those treated. While complete seizure freedom was not reported, seizures were shorter in duration, had reduced frequency, and were less intense. It is noted that many individuals who received VNS treatment had previous or current use of the ketogenic diet [27]. CDD patients with VNS were found to also have an earlier median age at the time of implantation, showing higher response with VNS implantation age of 6 years and younger [27,28]. A case report has outlined the electroclinical benefits of VNS in a CDD patient, showing high-voltage positive slow waves on interictal EEGs and improvement in psychomotor activity after VNS initiation [29].

VNS continues to be a good option in the range of treatments for patients with CDKL5 mutations. Further studies are needed to assess its long-term efficacy.

There are studies of CDD patients who have undergone a corpus callosotomy for seizure control showing reduction of seizures or complete resolution of seizures followed by recurrence (unspecified time period or within 1 year of surgery) [13]. Other studies have also shown that the surgery did not achieve more than a 50% reduction in seizure frequency [12]. 

More reports of individuals with CDD who have had a corpus callosotomy are needed to determine this surgery’s efficacy in reducing seizure burden.

In conclusion, for this refractory epileptic encephalopathy, CDD, many methods of treatment and seizure control have been trialed. This paper reviews the literature regarding the treatments of ganaxolone, fenfluramine, Epidolex, the ketogenic diet, and even surgical approaches. Of these methods, ganaxolone is the most promising for seizure control and was recently FDA-approved for CDD specifically. Fenfluramine demonstrates a good response but only for some of the seizure types characterized by CDD. Other methods demonstrated only 50% of patients responded to the ketogenic diet. Corpus collasotomy and VNS placement yield similar response rates; however, the limited power of studies reviewed prevents a full assessment of these methods on seizure control in CDD.

## Data Availability

Data sharing is not applicable to this article as no datasets were generated or analyzed during the current study.
